# Driving change or stuck in place? Mobility justice in Finnish and European transport policy

**DOI:** 10.1007/s13280-025-02283-w

**Published:** 2025-11-28

**Authors:** Emilia Suomalainen

**Affiliations:** Finnish Environment Institute, Latokartanonkaari 11, 00790 Helsinki, Finland

**Keywords:** Disadvantaged groups, Equity, Mobility justice, Road transport, Transport policy

## Abstract

While mobility justice has been the subject of much research, it is unclear to what degree the insights of the field are applied in national and EU-level transport policy. This work examines this question empirically by analysing transport strategies for the EU and Finland. The avoid–shift–improve framework is used to assess the effectiveness of the policy measures proposed for moving towards a more sustainable transport system. The results indicate that the justice dimension of sustainable mobility is not fully considered in transport policy and that car dependence and the inequalities caused by it are mostly referenced in an indirect way. While a shift to sustainable modes is advocated, there is a heavy focus on technological solutions. Electric vehicles and sustainable alternative fuels, the key technologies for transport decarbonisation, will, however, do little to reduce transport-related inequalities.

**Supplementary Information:**

The online version contains supplementary material available at 10.1007/s13280-025-02283-w.

## Introduction

Sustainable mobility aims to satisfy people’s basic transport needs to access places, opportunities, and services while respecting planetary limits and the principles of social justice. This definition, inspired by the imperatives defined by Holden et al. (2020), underlines that there is both an environmental and a justice dimension to sustainable mobility. Achieving sustainable mobility requires action beyond the transport sector: in addition to making all modes of transport more energy-efficient and environmentally friendly and encouraging the use of sustainable modes, it is necessary to implement various strategies aiming at the reduction of travel demand and distances. This threefold approach is commonly referred to as the avoid–shift–improve framework. In both the European and the Finnish contexts, moving towards sustainable mobility therefore implies transitioning from the current, heavily car-based transport systems towards more sustainable modes, namely walking, cycling, and public transport. This is an extremely challenging transformation, given that private cars are currently major providers of access. Indeed, in current transport systems improving access is often at odds with the environmental and justice pillars of sustainable mobility.

The unsustainability of current mobility systems is generally acknowledged. Transport is one of the largest sources of greenhouse gas (GHG) emissions in Europe, with road transport being the major contributor, generating over 76% of all transport GHG emissions, aviation and shipping included, at the EU level (European Environment Agency 2023). Private cars are responsible for over half of all road transport emissions in the EU (DG MOVE 2024). In addition to GHG emissions, road transport and cars in particular generate a multitude of other negative externalities (Miner et al. 2024). These externalities include air pollution, noise, and traffic crashes but also the depletion of natural resources, microplastics pollution, and land use and biodiversity impacts. These externalities often fall disproportionately on disadvantaged groups (Miner et al. 2024). In the context of urban mobility, injustice-related issues range from the exposure to negative externalities, such as traffic noise or air pollution, to distribution of space and to the prioritisation of different transport modes (Gössling 2016). Motorised transport is also linked to physical inactivity (Zukowska et al. 2022) that creates major health costs. In addition to a negative impact on liveability in urban areas, the adverse health impacts of transport bring major human as well as economic costs. Given our ‘car blindness’ (International Transport Forum 2024), these costs are to a large extent either ignored, seen as inevitable or, at best, the responsibility of individuals instead of a system-level issue.

Though accessibility is the focal point of transport systems planning and the environmental impacts of transport have long been acknowledged (see, for example, the EC Green Paper on transport externalities from 1992 (European Commission 1992), mobility justice has received less attention in transport policy (Ternes et al. 2024). While there is an increasing body of research (see, for example, Sheller (2018) on mobility justice and Martens (2016) on transport justice), I would argue that equity considerations are not often given the same weight in transport strategy and policy documents as the other two imperatives, accessibility and environmental impact. In transport system sustainability assessments, the social dimension is included only occasionally (Stefaniec et al. 2021). In terms of social impacts, safety- and access-related indicators are used much more frequently than equity indicators (Haghshenas and Vaziri 2012). Transport disadvantage, or difficulty to access places, opportunities, and services, is also linked to wider social exclusion (Lucas 2012). Transport poverty (Lucas et al. 2016) is another closely related concept.

This work aims to shed light on how transport-related inequalities are dealt with in strategic transport policy documents in the EU and in Finland. Recognising the strong links between car-oriented transport systems and mobility injustice, a major focal point of the investigation is whether the proposed policy measures can be thought to contribute to a transition towards a less car-centric and more just transport system. In recent years, there has been a movement towards creating more liveable and healthy cities (see, for example, Nieuwenhuijsen 2021)—this type of a shift can only be achieved by challenging the priority currently given to cars as a means of access.

## Materials and methods

I employ qualitative content analysis (Hsieh and Shannon 2005) to examine key transport strategy and policy documents. As a case study, this work focuses on the European Commission’s Sustainable and Smart Mobility Strategy (European Commission 2020) and Finland’s National Transport System Plan 2021–2032 (Ministry of Transport and Communications 2021a). This work examines (i) how the transport system is described with regard to its purpose and whether the positive and negative impacts (benefits and burdens) created by it are acknowledged. Secondly, I investigate (ii) whether there are any explicit mentions of fairness, justice, or equity. This includes mentions of disadvantaged, special needs, or vulnerable groups. Thirdly, (iii) the visions for a future transport system and their fairness implications are examined. Lastly, I analyse (iv) the potential justice implications of the proposed policy measures.

### Avoid–shift–improve framework

The avoid–shift–improve framework is used to categorise the different types of policy measures that are proposed for guiding development towards a more sustainable and less car-dependent mobility system. This framework was originally developed in Germany in the 1990s and reportedly appeared in a parliamentary report in 1994 (Creutzig et al. 2018; Leroutier and Quirion 2023). The framework has since been adopted in various sectors and used in a variety of applications (for an illustration of avoid–shift–improve options in different sectors, see Creutzig et al. (2018)). It is important to note that the three pillars of the avoid–shift–improve framework form a conceptual hierarchy, with ‘avoid’-type measures being the most fundamental (Jarre et al. 2024). Efficiency improvements (or ‘improve’-type actions)—such as improving the energy efficiency of engines or promoting the uptake of electric vehicles—should only come after demand for motorised transport has first been scaled down (‘avoid’) and shifted towards more sustainable modes, such as public transport and active travel (‘shift’). ‘Avoid’-type measures are typically focused on urban planning and proximity to physical but also to digital services and opportunities (e.g. remote working). In the transport context of the Global North, avoiding travel demand only applies to travel by motorised vehicles—active travel, notably walking and cycling, is to be encouraged. ‘Avoid’-type measures have recently been given visibility in Chapter 5 of IPCC’s AR6 where they are referred to as demand-side solutions (Creutzig et al. 2022).

### Content analysis

There were two major steps in the content analysis conducted in this work. Firstly, I familiarised myself with the case documents in question as well as with other relevant materials. Secondly, I systematically extracted references to issues related to mobility justice. In addition to paying attention to keywords such as ‘justice’, ‘fairness’, or ‘equity’, special attention was paid to text sections referencing different social groups (e.g. older people, people with reduced mobility or disabilities, and vulnerable road users). In addition, references to rural and peripheral areas were also examined. People living in rural areas are likely to experience difficulties due to high car dependence, and the rural context is often overlooked in transport policy (Flipo et al. 2023). References to transport externalities (air pollution, noise, congestion, etc.) were also scrutinised. A complete list of the findings can be found in Supplementary Information S1.

This methodology combines elements from conventional, directed, and summative content analysis (Hsieh and Shannon 2005). While the main method was conventional content analysis, the research for specific key words can be thought of as summative content analysis. The categorisation of policy measures using the avoid–shift–improve framework could be considered directed content analysis.

The terms ‘justice’, ‘fairness’, and ‘equity’ are used interchangeably in this work. The focal point is on distributive justice or the ‘fair distribution of benefits and burdens’ (Kaljonen et al. 2024), while the consideration of different population groups can be thought to refer to recognition justice (Sovacool et al. 2019a, b). For an overview of the different theories of justice underpinning the fields of mobility and transport justice, see, for example, Pereira et al. (2016) and Verlinghieri and Schwanen (2020). I also refer both to ‘transport’ and ‘mobility’, while acknowledging that ‘transport’ generally relates either to transport systems planning or the provision of transport services whereas ‘mobility’ is more linked to people and their needs.

A key assumption of the analysis is that the policy measures aimed at reducing car dependence positively contribute to mobility justice. Mattioli et al. (2020) define a car-dependent transport system as ‘one in which high levels of car use have become a key satisfier of human needs, largely displacing less carbon-intensive alternatives’. A particular focus is placed on the disadvantaged members of the carless population that suffer from the externalities of car-based mobility without enjoying its benefits in terms of access. Improving access for the carless and the disadvantaged could be argued to potentially improve access for everyone. While carelessness is a choice for some (the ‘carfree’), for others it is a constraint that negatively impacts their possibility to access places and opportunities (Karjalainen et al. 2023). On the other hand, car ownership can also be a financial strain, see Mattioli (2017) on ‘forced car ownership’.

The focus of this analysis is on everyday mobility. Long-distance trips and especially air travel, which has considerable equity implications (Gössling and Humpe 2020), fall outside the scope of the analysis.

### EU and Finland cases

The EU has extremely ambitious targets for reducing the climate impact of transport, aiming to cut transport emissions by 90% by 2050. This means that the pace of emission reductions in the sector will need to be fast—which might in turn create further tensions with regard to the (in)justice of the transition (Newell et al. 2022). Besides its own ambitious national goal of becoming carbon neutral by 2035, Finland is bound by EU targets and must reduce the emissions of its effort sharing sector—where transport is the biggest emitter—by 50% by 2030 compared to 2005 levels (Ministry of the Environment 2022).

In Finland, cars play a major role in the transport system: they are the main providers of access to jobs and services in large parts of the country and especially outside major urban centres (Traficom 2024a, 2024b, 2024c). This means that the lack of a motorised vehicle can easily lead to accessibility poverty, which is one form of transport poverty (Lucas et al. 2016). Even in urban areas in Finland more trips are undertaken by car than by any other mode, with the notable exception of the Helsinki region (Kallio et al. 2023). This car dependence has strong links to urban form: most of the housing in 20 Finnish city regions is in predominantly car-based zones (Finnish Environment Institute 2024), creating challenges for sustainable mobility for years to come.

Regional aspects are very important in national discussions in Finland: urbanisation is a megatrend, and outside the largest urban areas there is often a sentiment of their being ‘places that don’t matter’ (Mattila et al. 2020). Maintaining regional and peripheral accessibility is particularly challenging as Finland is a country with a relatively large surface area and low population density. While car-based access to regional centres remains good all over the country, those without a car at their disposal are at a disadvantage (Traficom 2024c). Ageing is also major trend (Statistics Finland 2024), and the situation of the ageing population is a growing concern in the country.

Two strategic documents on transport and mobility, an EU document and a Finnish one, were chosen as objects of analysis. The Sustainable and Smart Mobility Strategy of the European Commission (2020) is a strategic plan for reaching the ambitions of the European Green Deal or a 90% cut in transport emissions by 2050. This strategy covers all transport modes and proposes milestones as well as key areas of action or flagships. For Finland, the National Transport System Plan for 2021–2032 outlines the nation’s strategic vision for the development of the transport system over twelve years (Ministry of Transport and Communications 2021a). The National Transport System Plan also includes a more concrete action plan and a funding plan, but these were mostly left outside the scope to keep the analysis on a more strategic level. For this reason, I shall also refer to the National Transport System Plan as ‘a strategy’ when comparing the EU and Finland cases.

## Results

### EU’s Sustainable and Smart Mobility Strategy

#### Framing of and visions for the transport system

The vision of the Sustainable and Smart Mobility Strategy recognises the importance of mobility for economic and social life as well as the need to build a sustainable mobility system (European Commission 2020). It is acknowledged that in addition to its positive impacts, transport also brings many negative impacts on health and wellbeing, notably in the form of air pollution, noise, traffic crashes, and biodiversity impacts. The transition to a more sustainable mobility system is therefore also seen as a means towards a better quality of life.

It is recognised that sustainability and especially GHG emissions are a major challenge that must be solved in the transport sector. According to the vision, ‘The success of the European Green Deal depends on our ability to make the transport system as a whole sustainable’ (European Commission 2020). The aim of the strategy is to deliver a 90% reduction in transport emissions by 2050, in line with the Paris Agreement goals and EU’s climate neutrality ambitions. This goal of sustainability seems, however, somewhat at odds with the green growth narrative that appears early on in the document, where it is stated that ‘Greening mobility must be the new licence for the transport sector to grow’ (European Commission 2020).

#### References to justice

Several explicit mentions of fairness and justice are made in relation to the green transition of the mobility system. The transition to a zero-emission transport system is presented as an opportunity that will also serve future generations: ‘The EU has now an opportunity to build a mobility system that is sustainable, smart, and resilient: a system for future generations’ (European Commission 2020). It is stated that the future sustainable transport system must adapt and respond to users’ mobility needs and changing transport patterns, while ‘smart mobility’ with its advanced technology will provide ‘seamless, safe and secure connectivity to all European citizens’ (European Commission 2020). These changing mobility patterns are linked to the rise of digital solutions such as teleworking, videoconferencing, and e-commerce but also shared mobility.

The green transition is presented as a twin of the digital transition. At the same time, it is recognised that these twin transitions leading to a transformation of the transport system and the economy in general will not be easy, and it is underlined in the conclusion that the transformation needs to be ‘socially fair and just’ (European Commission 2020). It is also stated that ‘This evolution should leave nobody behind’: available and affordable mobility options are also needed in rural and remote areas and by persons with disabilities or reduced mobility (European Commission 2020).

Of the ten flagship areas, Flagship 9 or ‘Making Mobility Fair and Just for All’ explicitly targets mobility justice (European Commission 2020). Nevertheless, many of the other flagships and their milestones also have mobility justice implications. Under Flagship 9, ‘the need for affordable, accessible and fair mobility’ is highlighted (European Commission 2020). The crucial importance of justice is underlined: ‘The shift towards sustainable, smart and resilient mobility must be just or else risks not taking place’ (European Commission 2020). There are mentions of different groups currently experiencing transport disadvantages: people with low income (that find mobility expensive), people with disabilities or reduced mobility (that face accessibility challenges), and people living in rural, peripheral, and remote areas (that suffer from poor public transport connections). It is ‘essential to guarantee unhindered access to mobility for all’ (European Commission 2020).

#### Policy measures

The transition towards a sustainable mobility system requires system-level change, and the Sustainable and Smart Mobility Strategy proposes three pillars for action: (1) making all modes of transport more sustainable, (2) making sustainable alternatives more widely available, and (3) creating the right incentives for the transition (European Commission 2020). Different types of policy measures are given under these three pillars. Under the first pillar, there are measures to reduce dependence on fossil fuels. This means in practice replacing existing vehicles with new zero- and low-emission vehicles and increasing the uptake of renewable and low-carbon fuels. The second pillar prescribes a shift to more sustainable transport modes, especially rail. The third pillar focuses on the internalisation of external costs.

It is recognised in the strategy that the zero-emission technologies of the first pillar will not solve all sustainability issues; for example, electric cars will still produce noise and microplastics. It is also recognised that we need to continue to reduce air pollutant emissions. Under the second pillar, the aim is to allow better modal choices through more widely available sustainable, affordable, and fast travel alternatives: ‘People are willing to switch to more sustainable modes of transport, in particular in their daily mobility, with the main condition for switching being the cost, availability and speed’ (European Commission 2020). The EU can foster this modal shift by providing competitive sustainable alternatives that are available at ‘competitive prices, frequencies and comfort levels’ (European Commission 2020). In cities, cleaner and more active mobility is expected to contribute to the health and wellbeing of inhabitants. The connectivity of rural and suburban areas is also to be improved, and commuters are to be given more sustainable mobility options. The role of cities is underlined: ‘Cities are and should therefore remain at the forefront of the transition towards greater sustainability’ (European Commission 2020).

The third pillar concerns incentives for more sustainable transport choices. These incentives can be economic, such as carbon pricing, taxation, and infrastructure charges, or information-based, e.g. the carbon footprint of different travel options. It is recognised that modal choice plays a substantial role in the environmental footprint of transport but also in healthier mobility. It is stated that ‘The “polluter pays” and “user pays” principles need to be implemented without delay’ (European Commission 2020). The aim is the internalisation of external costs so that ‘those who use transport will bear the full costs rather than leaving others in our society to meet them’ (European Commission 2020). The goal is to put in place policies that produce ‘fair and efficient pricing’ in all transport modes and lower the cost of sustainable modes (European Commission 2020).

### Finland’s National Transport System Plan

#### Framing of the transport system and references to justice

In Finland’s National Transport System Plan, the transport system is framed as essential to the functioning of society (Ministry of Transport and Communications 2021a). The societal goals of the plan include fighting climate change and providing regional accessibility but also promoting Finland’s economic competitiveness. It is stated that ‘Development of the transport system is essential in terms of regional development and land use, business competitiveness, combating climate change, good everyday life, security of supply, etc.’ (Ministry of Transport and Communications 2021a). The freedom of movement and the freedom to engage in commercial activity, as granted by the Finnish Constitution, are also referenced. This latter reference is used to further underline the need to maintain and develop the transport system to serve business and commercial interests.

Finland is a country with a small population, 5.6 million inhabitants in 2023 (Statistics Finland 2024), and an extensive road network. The condition of the road network and the funding of its maintenance are central to many discussions of the national transport system. The state of the road network is also seen as an equity issue (Ministry of Transport and Communications 2021a): ‘The transport network’s condition and daily service levels have significant effects on society. … The condition of low-volume roads and railways has deteriorated.’ Congestion is mentioned as an issue on the access and ring roads in the largest urban sub-regions.

Regional equity is referenced in several parts of the plan. For instance, it is underlined that ‘Transport system planning is a means of advancing these societal goals while also responding to the various mobility and transport needs of customers, i.e. people and businesses, in different parts of Finland’ (Ministry of Transport and Communications 2021a). Peripheral areas are also explicitly mentioned: ‘Important business and commuting connections will be preserved between regional peripheries and regional and other key centres’ (Ministry of Transport and Communications 2021a). The freedom of movement granted by the Finnish Constitution is referenced several times, and it is stated that: ‘The plan’s measures will also improve people’s ability to choose different modes of mobility making use of various transport services’ (Ministry of Transport and Communications 2021a). The plan is therefore aiming to improve multimodality.

The negative externalities generated by transport are acknowledged in the light of the Constitution of Finland according to which ‘the public authorities must endeavour to guarantee for everyone the right to a healthy environment’ (Ministry of Transport and Communications 2021a). The plan then goes on to state that the goal is to promote the most environmentally sustainable modes of mobility, thus reducing the negative environmental impacts of transport.

The needs of vulnerable groups are explicitly mentioned in several sections. For instance, there is a reference to a UN Convention regarding people with disabilities and their right to access transportation on an equal basis. With respect to older people, the plan states: ‘The ageing population will influence traffic behaviour and mobility habits, which will need to be taken into account in transport system planning’ (Ministry of Transport and Communications 2021a). The action plan section of the document also references the special needs of different population groups and notably passenger transport services subsidised by the Social Insurance Institution of Finland, Kela. The development of these services from a client needs perspective is proposed: ‘Integrated passenger transport services provided through new types of cooperation could secure minimum passenger transport services, especially in sparsely populated areas’ (Ministry of Transport and Communications 2021a).

#### Visions for the future transport system

The plan acknowledges that climate change mitigation is a major challenge in which the transport sector plays a key role. Remarkably, it is also acknowledged that the country’s transport emissions are not significantly lower than in the 1990s and that: ‘In addition to choices of propulsion systems, cutting down transport emissions will particularly require reductions in passenger car traffic. However, passenger road transport is expected to continue growing at quite a steady rate’ (Ministry of Transport and Communications 2021a). There is thus a disconnect between the official transport demand forecasts and climate targets. While the need for technological solutions is underlined in various parts of the document, it is acknowledged that a reduction in car travel is necessary to meet climate targets (Ministry of Transport and Communications 2021a).

One of the key purposes of the National Transport System Plan is to propose a vision for the future transport system (Ministry of Transport and Communications 2021a): ‘In 2050, Finland’s transport system will function in a way that is environmentally, socially and economically sustainable, ensuring sufficient accessibility for people and businesses. Transport will function in a multimodal and emission-free manner. Mobility and logistics costs will have decreased.’ It is also expected that environmental harm caused by transport will decrease. In addition to environmental considerations, equity is addressed: ‘The transport system will be accessible and equal to all user groups’ (Ministry of Transport and Communications 2021a).

It is noted in the plan that almost 95% of the Finnish population lives close to urban centres and that urbanisation continues (Ministry of Transport and Communications 2021a). It is also acknowledged that the passenger car is the most common mode everywhere, except in Helsinki, the capital, where more trips are made on foot. For this reason, urban sub-regions are crucial in achieving sustainable mobility. For instance, commuters are expected to mainly use sustainable modes for trips both within and between urban sub-regions with public transport forming ‘the backbone of sustainable transport’ (Ministry of Transport and Communications 2021a). In particular, the development of rail is expected to structure and support sustainable urban development. The importance of both ‘avoid’ and ‘shift’ strategies is underlined, with knowledge workers shifting to working remotely and digital technologies enabling remote services. Active travel is expected to replace a considerable share of car trips in urban sub-regions. All in all, the development of multi- and intermodality is underlined: ‘Use of new versatile mobility and transport services will have become an established feature of everyday life’ (Ministry of Transport and Communications 2021a).

While the vision describes urban sub-regions as the ‘engines of economic growth while providing attractive living environments’, it is expected that those living in more sparsely populated areas will also have access to better mobility services (Ministry of Transport and Communications 2021a). In this case, however, ‘improve’ actions are underlined through low-emission passenger cars. The need to provide alternative fuels infrastructure enabling this low-emission mobility is not forgotten: ‘People will be able to use fossil-free propulsion systems throughout the country’ (Ministry of Transport and Communications 2021a).

#### Policy measures

The actions of the National Transport System Plan serve three high-level objectives (Ministry of Transport and Communications 2021a): (1) accessibility everywhere within the country (‘The transport system will ensure access to the whole of Finland and will respond to the needs of business, employment and housing’), (2) more opportunities for choosing sustainable modes, especially in urban areas, and (3) greater socioeconomic efficiency.

Regarding accessibility, different population groups are explicitly mentioned: ‘Mobility opportunities will be ensured for different population groups to guarantee social sustainability’ (Ministry of Transport and Communications 2021a). The role of the transport system in supporting sustainable urban form is given particular attention: ‘The transport network will only be expanded if this promotes sustainable [community] structures’ (Ministry of Transport and Communications 2021a).

Regarding sustainability, the plan states that sustainable modes will be promoted using a variety of means, especially in urban sub-regions but also elsewhere. The explicit aim is that the modal shares of active travel (walking and cycling) and public transport shall increase.

Regarding efficiency, it is stated that ‘The existing transport network will be put to maximum use while implementing the most efficient and effective measures to close any gaps’ (Ministry of Transport and Communications 2021a). The plan states that any new transport investment shall promote sustainable transport and that the social benefits of projects should be greater that their costs.

### Comparison of the EU and Finland cases

There are both similarities and differences in how issues related to mobility justice are treated in EU’s Sustainable and Smart Mobility Strategy and in Finland’s National Transport System Plan (Table 1). Both strategies make several references to justice, fairness, or equity: the Finnish strategy talks of a socially just transport system, while the EU strategy states that the transformation of the transport system should be ‘socially fair and just’. The justice dimension is also underlined in the EU strategy via Flagship 9 or ‘making mobility just and fair for all’.

**Table 1:** Comparison of the EU and Finland cases for selected mobility justice-related issues. ^a^European Commission (2020). ^b^Ministry of Transport and Communications (2021a)

Item	EU case^a^	Finland case^b^
References to a just transition or social justice	‘this [green and digital] transformation … needs to be socially fair and just’	Several references to social sustainability
Vulnerable road users	‘Protecting vulnerable road users will be a priority’	No explicit reference, but: ‘Traffic safety will be at a high level in all modes of transport and no-one will need to die or sustain serious injuries in traffic.’
Peripheral, rural, and remote areas	Several references to rural areas, notably: ‘In rural, peripheral and remote areas, including the outermost regions and islands, improved public transport links will be essential to guarantee unhindered access to mobility for all.’	Differentiation between urban sub-regions and more peripheral areas: ‘Sustainable modes of mobility and transport will also be developed outside urban sub-regions.’
Older people	No references	Acknowledgement that ageing will need to be taken into account in transport system planning; reference to ‘accessible services’ for the ageing
People with challenges using digital tools	Acknowledgment that mobility is not sufficiently accessible for those with poor IT literacy	No references
Gender and women	A reference to ‘equality mainstreaming’ and increasing the number of women in transport professions	No references
People with disabilities or reduced mobility	A reference to the just transition mechanism; also ‘it is crucial that mobility is available and affordable for all, that rural and remote regions are better connected, accessible for persons with reduced mobility and persons with disabilities’	A reference to a UN convention on equal access; focus on mobility services: ‘mobility services are developing towards an increasing variety of options, offering all user groups flexible, efficient, accessible and low-emission mobility services.’
Indigenous groups	No references	‘The Saami people’s rights and needs must be taken into account in transport system development in Northern Finland.’
Low-income population	Affordability referenced multiple times, including: ‘it is crucial that mobility is available and affordable for all’	Vision of a decrease in mobility costs
Carless population	No explicit references, but: ‘It is crucial that mobility is available and affordable for all’	No explicit references, but: ‘Mobility opportunities will be ensured for different population groups to guarantee social sustainability.’
High emitters (especially high-income groups)	No references	No references
Global justice	References to the lifecycle impacts of vehicles and their batteries	No references

Both in the EU strategy and in the Finnish plan, there is little explicit acknowledgement of our current car dependence and very car-centric transport systems (Table 2). There are few explicit mentions of reducing car travel in the EU strategy, except in terms of congestion, while collaborative and shared mobilities are expected to reduce the number of vehicles on the road. While congestion and car dependence are related, car dependence is a much larger issue. There is, however, an explicit mention of the necessity to reduce passenger car travel in the Finnish strategy.

**Table 2:** Comparison of the EU and Finland cases for selected car dependence-related issues. ^a^European Commission (2020). ^b^Ministry of Transport and Communications (2021a)

Item	EU case^a^	Finland case^b^
Reduction of car use and mileage	No explicit references, but shared and collaborative mobility services are seen as a way to reduce congestion; also ‘it is also imperative to address the cost of pollution and congestion for society’	An explicit acknowledgement of the need to reduce car use and of the fact that this is in contradiction with official projections: ‘In addition to choices of propulsion systems, cutting down transport emissions will particularly require reductions in passenger car traffic. However, passenger road transport is expected to continue growing at quite a steady rate.’
Reduction of car ownership	No explicit references, but several mentions of shared and collaborative mobility services	No explicit mentions, but several references to mobility services
Mode shift to active travel and public transport	The second pillar focuses on a shift to more sustainable modes ‘The EU must help create appropriate conditions for the higher uptake of sustainable alternatives that are safe, competitive and affordable. Where suitable alternatives are in place at competitive prices, frequencies and comfort levels, people choose the more sustainable mode.’	‘The shares of public transport, walking, cycling and other sustainable modes of mobility will increase while greenhouse gas emissions from transport will decrease, contributing to the achievement of the climate target.’
Sustainability of new infrastructure investments (e.g. road building)	No references	‘New transport investments will promote sustainable transport and their social benefits will exceed the costs involved.’; ‘The transport network will support and promote sustainable community structures. The transport network will only be expanded if this promotes sustainable structures.’
External costs of transport	‘[Mobility] is not without costs for our society. These include greenhouse gas emissions, air, noise and water pollution, but also accidents and road crashes, congestion, and biodiversity loss—all of which affect our health and wellbeing. Past efforts and policy measures have not yet sufficiently addressed these costs’; several references to the ‘polluter pays’ principle; milestone: ‘All external costs of transport within the EU will be covered by the transport users at the latest by 2050.’	‘New transport investments will promote sustainable transport and their social benefits will exceed the costs involved.’
Externalities of car use	Acknowledgement that EVs will not solve all issues, e.g. microplastics and noise; also a reference to new air pollution standards	No explicit references, but a reference to the constitution and the right to a healthy environment
Benefits of reducing car travel (health, wellbeing, livability, etc.)	‘by cleaner and more active mobility in greener cities that contribute to the good health and wellbeing of their citizens’; Flagship 3: ‘Making inter urban and urban mobility more sustainable and healthy’	No references

In Finland’s National Transport System Plan, the shift to sustainable modes of transport is to be promoted in the urban sub-regions whereas elsewhere the focus is more heavily on electrification and improving efficiency. Nevertheless, those living in sparsely populated areas are also expected to have access to better mobility services, though it is unclear how. Importantly, the close link between land use and sustainable transport is recognised, as urban sprawl both creates and perpetuates car dependence (Mattioli et al. 2020). In Finland, this aspect is tackled in practice through land use, housing, and transport agreements between the state and the largest city regions. While rural and remote areas are also mentioned in the EU strategy with ‘unhindered access to mobility for all’ (European Commission 2020), it is difficult to see how public transport could be made available for all, except at considerable cost.

The EU strategy refers to the affordability and costs of mobility multiple times. This is not the case in Finland’s transport system plan, although its vision describes a decrease in mobility and logistics costs. In Finland, the focus is very strongly on cost efficiency from the transport system planning perspective. Both strategies acknowledge the need to reduce the external costs of transport, with the EU strategy referring multiple times to the polluter pays principle, internalisation of external costs, and carbon pricing. A milestone aims at the internalisation of all external costs of transport by 2050. While an excellent idea, this leaves open many questions as to what external costs of transport are to be taken into account and how they are to be estimated and translated into a monetary value. Cost–benefits assessment methodologies used in the transport sector have been criticised given that they systematically underestimate the costs of automobility (Gössling et al. 2019).

The case of high emitters and especially high-income groups producing a disproportionate share of transport emissions is not addressed in either strategy (for an analysis of high emitters, see, for example, Mattioli et al. 2023). This has potential implications for the acceptability of policy measures impacting those at lower income levels. Transport is a sector with a great amount of inequality in carbon footprints both within and between nations (Ivanova and Wood 2020; Oswald et al. 2020). Price-based incentives might also be ineffective for higher income groups, unless directly linked to the income level. From a justice perspective, the case of high-income groups is crucial, as they would need to reduce their emissions the most, both in absolute and in relative terms.

While health and wellbeing are mentioned several times in the EU strategy, the benefits of reducing car use and increasing active travel could be underlined further. It has been shown that motorised travel and especially car use generates major societal costs (Gössling et al. 2019; Pisoni et al. 2022). Finland’s transport system plan does not mention health and liveability in its strategic part although these benefits are acknowledged in the action plan on walking and cycling infrastructure.

The EU strategy underlines that the green and digital transitions ‘should leave nobody behind’ while the potential issues linked to digitalisation, notably for people who have difficulties using IT tools, are acknowledged (European Commission 2020). It is somewhat surprising that the potential issues linked to increasing digitalisation are not discussed in Finland’s National Transport System Plan given that ageing is a megatrend in Finland. In the Finnish strategy, the needs of older people are mentioned; this reference is, however, framed in terms of accommodating their behaviours and habits, rather than in terms of specific needs and capabilities.

All in all, it seems unlikely that the proposed policy measures will be sufficient to produce systemic change, the necessity of which is underlined in the EU vision. Both strategies are heavily focused on actions such as electrification, sustainable alternative fuels, efficiency, and digitalisation. The problem regarding mobility justice is that ‘improve’-type actions will not significantly benefit the most disadvantaged and vulnerable groups and the carless population in general. In both strategies, new mobility services contributing to multimodality are seen as promoting a shift to more sustainable transport modes and improving accessibility for different social groups. There is a heavy emphasis on these new mobility services—but are they truly the panacea they are made out to be?

## Discussion

### Contributions to practice

According to the principles of the avoid–shift–improve framework, the strategic priority should be given to ‘avoid’ actions, or reducing travel demand and distances, while remaining demand should be directed towards the most sustainable modes. Lastly, the negative impacts (e.g. emissions, pollution, and energy and resource use) of all transport modes should be minimised.

One of the key justice issues in the EU’s mobility strategy and Finland’s transport system plan is that the emphasis is on ‘improve’ actions; the focus on electric vehicles (EVs) and renewable fuels could be argued to take much-needed attention away from the reduction of motorised transport. This emphasis on zero-emission vehicles and sustainable alternative fuels might even further bolster the car-dependent status quo through new investments. The EU strategy, in particular, is very technology-driven. While zero-emission vehicles and low-carbon fuels are no doubt necessary for reducing transport emissions, they are not sufficient and create new types of environmental and social impacts (see, for example, Sovacool et al. 2019a, b; Dall-Orsoletta et al. 2022; Hosseini and Stefaniec 2023). The EU strategy recognises that the electrification of the car fleet will not solve all its environmental issues, such as non-exhaust emissions, noise, or pollution from microplastics. In the case of biofuels, there is a risk that the emission reductions obtained in the transport sector create negative environmental impacts elsewhere, in other sectors and countries, as the production of biofuels can have negative land use impacts and contribute to the loss of forest sinks (Soimakallio and Koponen 2011; Koponen et al. 2013; Koponen and Soimakallio 2015). This carbon leakage is also a concern for electrification which transfers environmental and emission impacts from countries where EVs are used towards countries where their manufacturing and raw materials extraction takes place (Dall-Orsoletta 2022).

Increasing EV demand will also lead to an increasing demand for various critical or strategic raw materials, such as cobalt and lithium, and a greater material footprint (Sen et al. 2019; Jones et al. 2020). As with all mining, the extraction of these raw materials is not without environmental impacts. In addition to EVs themselves, the production of renewable electricity necessary to power these vehicles and energy storage systems also require critical raw materials (Calvo and Valero 2022). The EU strategy, at least implicitly, acknowledges that electrification is transferring impacts towards countries where manufacturing and raw material extraction takes place, as there are several mentions of lifecycle impacts and actions to reduce them.

The ‘shift’ actions proposed in these transport strategies are underdeveloped. Both the EU strategy and Finland’s plan rightly underline the role of cities as frontrunners in sustainable mobility. One of the flagships of the EU strategy focuses on the provision of sustainable alternatives in urban and interurban mobility: it is thought that if sustainable alternatives are available at competitive prices, frequencies, and comfort levels people will choose them (European Commission 2020). The reality might not be this simple, as there are many subjective factors that impact car ownership and use (Soza-Parra and Cats 2023). It is also not clear as to how competitive prices, frequencies, and comfort levels should be defined—both active travel and public transport are fundamentally different from car use in many aspects (exposure to the elements, timetable constraints, privacy, speed, etc.). While a shift to more sustainable modes is prescribed by both strategies, we are unlikely to see significant changes unless driving is at the same time made less attractive (Buehler et al. 2017; Xiao et al. 2020; Kuss and Nicholas 2022).

The necessity to undertake ‘avoid’-type actions to reduce car use is only acknowledged in the Finnish case. The EU strategy relies on a green growth narrative, decoupling the environmental impacts of transport from the sector’s expected growth. While this might be feasible in term of climate impacts, it is difficult to see how many other environmental impacts might be negated without a substantial reduction in car-based mobility. It is acknowledged in the EU strategy that zero-emission vehicles will not solve or will only partially solve non-climate environmental issues linked to car use, notably noise and air pollutant emissions. One proposed solution is distance-based road charging, which could help regulate the mileage driven and alleviate environmental issues that remain after a transition to electric mobility. It is, however, acknowledged that ‘Substantial progress is needed on effective charging for infrastructure use’ (European Commission 2020). Contrary to many other countries, Finland currently has no road use-based charges.

Finland’s National Transport System Plan, for its part, heavily underlines the economic rationales of transport. The needs of businesses as well as of inhabitants are used to justify the maintenance of an extensive (and costly) road network over the national territory. On the other hand, the plan recognises the need to cut down car use and GHG emissions to meet climate targets. Past failures in reducing emissions in the transport sector are candidly acknowledged. Remarkably, while there is an objective to stabilise the mileage of the Finnish passenger car fleet (Ministry of Transport and Communications 2021b), national transport demand forecasts depict a growth both in the national car fleet and in its mileage (Moilanen et al. 2024). While there is no explicit commitment to stop road building, new projects should support sustainable urban development (Ministry of Transport and Communications 2021a). It is unclear as to how sustainability is defined in this context, and it is equally unclear whether and how the sustainability of new road investment projects included in the funding plan has been justified.

### Contributions to theory

This study highlights the usefulness of employing the avoid–shift–improve framework in policy analysis: the framework provides a conceptual hierarchy to analyse which policy measures have the most potential to effectively reduce car dependence. The utility of this framework has also recently been demonstrated in the energy sector, where Jarre et al. (2024) showed that ‘avoid’-type energy policies are underrepresented. While ‘avoid’ measures also seem to garner relatively little attention in passenger road transport, it is possible that this finding is linked to the scope of the documents examined. Transport and mobility strategies tend to be, by definition, limited to the transport sector, whereas ‘avoid’ measures typically go beyond, involving land use and urban planning, provision of essential services, and digital connectivity. The concept of triple access planning developed in the UK—linking the transport system, land use planning, and telecommunications—makes this link between mobility, proximity, and digital access explicit (Triple Access Planning for Uncertain Futures 2025).

In their analysis of the political economy of car dependence, Mattioli et al. (2020) assert that the political and economic underpinnings linking human needs satisfaction to energy consumption (and its impacts) are often ignored. They identify five constituents of car-dependent transport systems: (i) the automotive industry, (ii) the provision of car infrastructure, (iii) the political economy of urban sprawl, (iv) the provision of public transport, and (v) cultures of car consumption (Mattioli et al. 2020). Mattioli et al. (2020) also underline that in order to move away from car dependence all these five interconnected elements will need to be tackled. The first and last of these elements, the automotive industry and car cultures, fall outside the traditional scope of the avoid–shift–improve framework. This means that to cover all these factors, the scope of ‘avoid’-type actions would need to be extended to economic and industrial as well as to cultural directions. Another option would be to add another contextual level covering diverse economic and cultural factors. Another useful framework might be the three spheres of social transformation (practical, political, and personal) proposed by O’Brien (2018).

While the types of high-level strategic documents analysed in this work are no doubt central, it is unclear as to what extent their contents get translated into concrete actions implemented on the ground. For instance, both in the EU and in Finland city regions often have their own mobility plans and strategies, for instance in the form of Sustainable Urban Mobility Plans (SUMPs). In the future, it would be interesting to have a closer look at the local level to see if the traits found at the EU and national levels are reflected there or if city regions might be considered more progressive regarding mobility justice. In North America, it was found that urban transportation plans often failed to translate equity-related goals into measurable objectives and that appropriate means for measuring progress were lacking (Manaugh et al. 2015). Inequality issues also often arise at the implementation stage, despite discourses of sustainability. Boussauw and Vanoutrive (2017) showed using Belgian examples that actions implemented to improve sustainability can also lead to unsustainable outcomes and social injustice.

One of the shortcomings of an approach focused on analysing mobility justice implications through measures reducing car dependence is that these two goals—greater equity and lesser car dependence—might in some (rare) cases be contradictory. This can notably be related to how high-level ideas are translated into concrete action (see, for example, Boussauw and Vanoutrive (2017)) or stem from the details of implementation. I would like to acknowledge that there are use cases where a (low-emissions) car remains the only feasible travel option for the foreseeable future either because of distances or other factors (travel during nighttime, transport of heavy equipment, time-critical services, etc.). It would be useful to identify these hard-to-avoid and hard-to-shift use cases where ‘improve’ actions are most direly needed.

In an analysis of the social sustainability of road transport in EU member states Finland had one of the highest social sustainability scores among the EU-15 (Stefaniec et al. 2021). Stefaniec et al. (2021) focused only on social factors so as not to distort the results by including economic indicators; the countries with the highest car ownership rates were found to score lower on social sustainability, and the correlation was even stronger when considering the number of cars in relation to the extent of the road network. Finland is an interesting case as it has a high rate of car ownership in EU statistics (DG MOVE 2024). It is possible that the high social sustainability performance is explained by the fact that the country has an extensive road network for relatively few inhabitants (due to a low population density and a relatively large national territory)—and the authors acknowledge that the road network is a proxy for potential rather than actual access (Stefaniec et al. 2021). This underscores the need to define measurable car dependency and/or equity-related indicators that might be used to assess desirable or undesirable outcomes in quantitative assessments of social sustainability.

The focus of this analysis was on car dependence, the transition to more sustainable mobility, and the related equity implications. It is crucial to tackle car centricity in a way that improves mobility justice and does not, for instance, create insurmountable difficulties for low-income car-dependent people or others who experience forced car ownership. (For an analysis of the economic equity implications of different policies for reducing car use see Lindsey et al. (2023).) The key questions are: who should reduce car use, where, and on which trips? While an emphasis is placed on city regions where more alternatives are available, the case of low-density areas is especially difficult. In rural areas and on the urban–rural fringe, there are fewer options to the car, and the yearly mileage is generally higher than in urban centres. While the case of peripheral, rural, and remote locations is mentioned several times in both the EU and Finnish strategies, it remains unclear what sustainable mobility would look like in practice at these locations.

## Conclusion

In this work, I analysed EU’s Sustainable and Smart Mobility Strategy and Finland’s National Transport System Plan to (i) examine the framing of the transport system, in particular its purpose; (ii) find any explicit mentions of fairness, justice, or equity; (iii) examine the visions for future; and (iv) analyse the potential mobility justice implications of proposed policy measures. Particular attention was paid to the inequalities created by car dependence, and the avoid–shift–improve framework was used to structure the analysis. The analysis shows that while Finland’s National Transport System Plan recognises the necessity to reduce car mileage, the EU document only refers to battling congestion. In Finland, issues linked to ageing and regional equity are acknowledged given their great societal importance. Generally speaking, in both cases the focus of transport policies seems to be, firstly, on ‘improve’ actions, especially electrification and sustainable alternative fuels, while the ‘shift’ to more sustainable modes of transport comes second. The reduction of travel demand, or ‘avoid’, only comes third and is mostly linked to digitalisation. Given this order of priorities, it is worrisome that the justice and equity implications of electrification are not discussed. As the problem of car dependence is not named in these strategic documents, it is also doubtful that the proposed policy measures will succeed in shifting the car-centric status quo towards a more sustainable as well as a more just mobility system.

## Supplementary Information

Below is the link to the electronic supplementary material.

Supplementary file1 (PDF 747 KB)

## Data Availability

The documents analysed during this study are publicly available and referenced in the article. The data generated in the course of the study are available in the Supplementary Information file.
