# Supplementary material for: Driving change or stuck in place? Mobility justice in Finnish and European transport policy
Source: Ambio. 2025 Nov 28;55(9):2049–62. doi: 10.1007/s13280-025-02283-w (PMC13438032; doi:10.1007/s13280-025-02283-w)
Supplement: Supplementary file 1 [file 13280_2025_2283_MOESM1_ESM.pdf]

Supplementary Information: This Supplementary Information has not been peer reviewed.
Title: Driving change or stuck in place? Mobility justice in Finnish and European transport policy

Table S1 Mentions of issues related to justice and equity in EU's Sustainable and Smart Mobility Strategy

Section	Page	Paragraph, flagsip, or milestone	Citation [1]	Category	Keywords
3 SUSTAINABLE MOBILITY – AN IRREVERSIBLE SHIFT TO ZERO-EMISSION MOBILITY	3	11	This implies that all policy levers must be pulled: (1) measures to significantly reduce the current dependence on fossil fuels (by replacing existing fleets with low- and zero-emission vehicles and boosting the use of renewable and low-carbon fuels); (2) decisive action to shift more activity towards more sustainable transport modes (notably increasing the number of passengers travelling by rail and commuting by public transport and active modes, as well as shifting a substantial amount of freight onto rail, inland waterways, and short sea shipping); and (3) internalisation of external costs (by implementing the 'polluter pays' and 'user pays' principles, in particular through carbon pricing and infrastructure charging mechanisms).	policy measures/actions	zero-emission vehicles, low-carbon fuels, mode shift, carbon pricing, external costs, polluter pays
2.1 We need to make all modes of transport more sustainable	3	12	As the first pillar of our approach, we must boost the uptake of low- and zero-emission vehicles as well as renewable and low-carbon fuels for road, waterborne, air and rail transport, without further delay. [...] Maintaining technology-neutrality across all modes is key, but this should not lead to inaction on eliminating fossil fuel-based solutions.	policy measures/actions	zero-emission vehicles, low-carbon fuels
2.1 We need to make all modes of transport more sustainable	3	Flagship 1	FLAGSHIP 1 – BOOSTING THE UPTAKE OF ZERO-EMISSION VEHICLES, RENEWABLE & LOW-CARBON FUELS AND RELATED INFRASTRUCTURE	policy measures/actions	zero-emission vehicles, low-carbon fuels
2.1 We need to make all modes of transport more sustainable	4	15	The Commission will propose actions to boost the uptake of zero-emission vehicles in corporate and urban fleets.	policy measures/actions	zero-emission vehicles
2.1 We need to make all modes of transport more sustainable	7	Milestone 2	By 2050, nearly all cars, vans, buses as well as new heavy-duty vehicles will be zero-emission.	policy measures/actions	zero-emission vehicles
3 SMART MOBILITY – ACHIEVING SEAMLESS, SAFE AND EFFICIENT CONNECTIVITY	20	100	Protecting vulnerable road users will be a priority [...] The upgrading of existing high-risk infrastructure should remain a priority for infrastructure investments, with a particular attention on ageing and underdeveloped network segments. Measures to give more space to various forms of active mobility will help prevent deaths and serious injuries for vulnerable road users.	policy measures/actions	vulnerable road users, road safety

2.2 We need to make sustainable alternatives widely available to enable better modal choices	8	37	Clearer guidance is needed on mobility management at local and regional level, including on better urban planning, and on connectivity with rural and suburban areas, so that commuters are given sustainable mobility options.	policy measures/actions	urban planning, cities, rural areas, commuting
1 OUR VISION	2	9	Overall, we must shift the existing paradigm of incremental change to fundamental transformation.	vision	system change
3 SMART MOBILITY – ACHIEVING SEAMLESS, SAFE AND EFFICIENT CONNECTIVITY	20	Flagship 10	FLAGSHIP 10 – ENHANCING TRANSPORT SAFETY AND SECURITY	policy measures/actions	road safety
3 SMART MOBILITY – ACHIEVING SEAMLESS, SAFE AND EFFICIENT CONNECTIVITY	23	Milestone 14	By 2050, the death toll for all modes of transport in the EU will be close to zero.	policy measures/actions	road safety
3 SMART MOBILITY – ACHIEVING SEAMLESS, SAFE AND EFFICIENT CONNECTIVITY	13	74	The EU has now an opportunity to build a mobility system that is sustainable, smart, and resilient: a system for future generations.	policy measures/actions	resilience, future generations
1 OUR VISION	2	6	Mobility in Europe should be based on an efficient and interconnected multimodal transport system, for both passengers and freight, enhanced by an affordable high-speed rail network, by abundant recharging and refuelling infrastructure for zero-emission vehicles and supply of renewable and low-carbon fuels, by cleaner and more active mobility in greener cities that contribute to the good health and wellbeing of their citizens.	vision	multimodality, zero-emission vehicles, low-carbon fuels, health, wellbeing
2.2 We need to make sustainable alternatives widely available to enable better modal choices	7	28	As a second pillar of our approach, sustainable alternatives must be made widely available now in a fully integrated and seamless multimodal mobility system. The EU cannot rely exclusively on technological solutions: immediate action to adapt our mobility system is necessary to tackle climate change and reduce pollution.	policy measures/actions	multimodality

2.2 We need to make sustainable alternatives widely available to enable better modal choices	8	31	Decisive action is needed to transform the transport sector into a truly multimodal system of sustainable and smart mobility services. To achieve this, Europe should build a high quality transport network with high-speed rail services on short-haul distances [...]	policy measures/actions	multimodality
2.2 We need to make sustainable alternatives widely available to enable better modal choices	8	35	As set out in the 2030 climate target plan, increasing the modal shares of collective transport, walking and cycling, as well as automated, connected and multimodal mobility will significantly lower pollution and congestion from transport, especially in cities and improve the health and well-being of people. Cities are and should therefore remain at the forefront of the transition towards greater sustainability. [...] all large and medium-sized cities that are urban nodes on the TEN-T network put in place their own sustainable urban mobility plans by 2030. The plans should include new goals, for example on having zero emissions and zero road fatalities.	policy measures/actions	mode shift, health, wellbeing, cities, road safety
2.2 We need to make sustainable alternatives widely available to enable better modal choices	8	29	The EU must help create appropriate conditions for the higher uptake of sustainable alternatives that are safe, competitive and affordable. Where suitable alternatives are in place at competitive prices, frequencies and comfort levels, people choose the more sustainable mode.	policy measures/actions	mode shift, affordability
2.1 We need to make all modes of transport more sustainable	4	17	The evolution of road vehicle engines towards zero emission does not as such solve issues raised by the use of tyres, which still cause noise and microplastics.	externalities	microplastics, noise
2.1 We need to make all modes of transport more sustainable	4	15	In addition, the new regulation on batteries will ensure that batteries placed on the EU market are sustainable and safe all along their entire life cycle. Sustainability and end-of-life cycle requirements, including on carbon footprint and ethical and sustainable sourcing of raw materials, are essential to reduce environmental footprint of electric vehicles.	externalities	life cycle emissions, global impacts
2.1 We need to make all modes of transport more sustainable	4	17	The upcoming revision of the Directive on end-of life vehicles will also aim at reducing the overall environmental footprint of the production and dismantling of cars.	externalities	life cycle emissions

1 OUR VISION	1	2	Whilst mobility brings many benefits for its users, it is not without costs for our society. These include greenhouse gas emissions, air, noise and water pollution, but also accidents and road crashes, congestion, and biodiversity loss – all of which affect our health and wellbeing. Past efforts and policy measures have not yet sufficiently addressed these costs. The transport sector's greenhouse gas emissions have increased over time and represent now as much as a quarter of the EU's total.	purpose/framing	impacts, emissions, health, wellbeing
2.2 We need to make sustainable alternatives widely available to enable better modal choices	8	Flagship 3	FLAGSHIP 3 – MAKING INTERURBAN AND URBAN MOBILITY MORE SUSTAINABLE AND HEALTHY	policy measures/actions	health
1 OUR VISION	2	6	Greening mobility must be the new licence for the transport sector to grow.	vision	green growth
2.2 We need to make sustainable alternatives widely available to enable better modal choices	12	50	Substantial progress is needed on effective charging for infrastructure use, notably in road transport. This is key to internalising the cost of damage to infrastructure, but it is also imperative to address the cost of pollution and congestion for society.	externalities	external costs, congestion, pollution
2.2 We need to make sustainable alternatives widely available to enable better modal choices	13	Milestone 10	All external costs of transport within the EU will be covered by the transport users at the latest by 2050.	externalities	external costs
1 OUR VISION	1	3	The success of the European Green Deal depends on our ability to make the transport system as a whole sustainable.	vision	European Green Deal
1 OUR VISION	1	3	By far, the most serious challenge facing the transport sector is to significantly reduce its emissions and become more sustainable. At the same time, this transformation offers great opportunities for better quality of life, and for European industry across the value chains to modernise, create high-quality jobs, develop new products and services, strengthen competitiveness and pursue global leadership as other markets are moving fast towards zero-emission mobility.	vision	emissions, transformation

2 SUSTAINABLE MOBILITY – AN IRREVERSIBLE SHIFT TO ZERO-EMISSION MOBILITY	3	10	The European Green Deal calls for a 90% reduction in greenhouse gas emissions from transport, in order for the EU to become a climate-neutral economy by 2050, while also working towards a zero-pollution ambition. To achieve this systemic change, we need to (1) make all transport modes more sustainable, (2) make sustainable alternatives widely available in a multimodal transport system and (3) put in place the right incentives to drive the transition.	policy measures/actions	emissions, European Green Deal, climate neutrality
2.2 We need to make sustainable alternatives widely available to enable better modal choices	8	30	At the same time, mobility patterns and consumer behaviour are changing. [...] Teleworking, video-conferencing, electronic commerce, the uptake of shared and collaborative mobility services, all contribute to the ongoing transformation of mobility.	vision	digitalisation, teleworking
2.2 We need to make sustainable alternatives widely available to enable better modal choices	8	36	Seamless multimodality enabled by digital solutions is vital in urban and sub-urban areas. Increasing pressure on passenger transport systems has boosted demand for new and innovative solutions, with various transport services being integrated into a service accessible on demand, following the Mobility as a Service (MaaS) concept. Simultaneously, many cities are witnessing a shift towards shared and collaborative mobility services (shared cars, bikes, ride-hailing, and other forms of micromobility) facilitated by the emergence of intermediary platforms, thereby enabling the reduction of the number of vehicles in daily traffic.	policy measures/actions	digitalisation, multimodality, MaaS, shared mobility, congestion
3 SMART MOBILITY – ACHIEVING SEAMLESS, SAFE AND EFFICIENT CONNECTIVITY	13	56	The EU needs to take full advantage of smart digital solutions and intelligent transport systems (ITS). Connected and automated systems have enormous potential to fundamentally improve the functioning of the whole transport system and contribute to our sustainability and safety goals.	policy measures/actions	digitalisation, ITS, automation
6 CONCLUSIONS	24	109	The recovery from the crisis caused by the COVID-19 pandemic should be used to accelerate the decarbonisation and modernisation of the entire transport and mobility system, limiting its negative impact on the environment and improving the safety and health of our citizens. The twin green and digital transitions should reshape the sector, redraw connectivity and re-energise the economy. The Commission acknowledges that this transformation – which needs to be socially fair and just – will not come easily [...]	justice	digitalisation, health, road safety

3 SMART MOBILITY – ACHIEVING SEAMLESS, SAFE AND EFFICIENT CONNECTIVITY	13	54	People should enjoy a seamless multimodal experience throughout their journey, through a set of sustainable mobility choices, increasingly driven by digitalisation and automation.	vision	digitalisation, automation, multimodality
3 SMART MOBILITY – ACHIEVING SEAMLESS, SAFE AND EFFICIENT CONNECTIVITY	13	Flagship 6	FLAGSHIP 6 – MAKING CONNECTED AND AUTOMATED MULTIMODAL MOBILITY A REALITY	policy measures/actions	digitalisation, automation, multimodality
1 OUR VISION	2	7	Digitalisation will become an indispensable driver for the modernisation of the entire system, making it seamless and more efficient.	vision	digitalisation
1 OUR VISION	1	4	The crisis has shown the essential role played by transport and the social, health and economic costs when free movement of people, goods and services is severely constrained or even curtailed altogether. The preservation of supply chains and a coordinated European approach to connectivity and transport activity are essential to overcome any crisis and strengthen the EU's strategic autonomy and resilience.	vision	Covid-19, resilience
1 OUR VISION	1, 2	5	Therefore, ensuring that our transport system is truly resilient against future crises must also be a key objective of the EU's transport policy going forward. [...] In the context of the recovery from this severe crisis, public support should help mobility "build back better" and leap forward to a sustainable and smarter future.	vision	Covid-19, resilience
2.1 We need to make all modes of transport more sustainable	4	14	Significant efforts have been made over the last 5 years to reduce emissions of air pollutants from motor vehicles. [...] But more can be done: the upcoming proposal for more stringent air pollutant emissions standards for combustion engine vehicles (Euro 7) will ensure that only future-proof low-emission vehicles come to the market.	externalities	air pollution
1 OUR VISION	2	8	This evolution should leave nobody behind: it is crucial that mobility is available and affordable for all, that rural and remote regions are better connected, accessible for persons with reduced mobility and persons with disabilities, and that the sector offers good social conditions, reskilling opportunities, and provides attractive jobs.	justice	affordability, rural areas, persons with disabilities

3 SMART MOBILITY – ACHIEVING SEAMLESS, SAFE AND EFFICIENT CONNECTIVITY	20	88	The economic shock has highlighted the need for affordable, accessible and fair mobility for passengers and other users of transport services. Indeed, whereas the single market in transport has increased connectivity, mobility remains expensive for people with low disposable income, and not sufficiently accessible for people with disabilities or reduced mobility, and those with low IT-literacy. In rural, peripheral and remote areas, including the outermost regions and islands, improved public transport links will be essential to guarantee unhindered access to mobility for all.	justice	affordability, rural areas, people with disabilities, reduced mobility
3 SMART MOBILITY – ACHIEVING SEAMLESS, SAFE AND EFFICIENT CONNECTIVITY	20	89	The shift towards sustainable, smart and resilient mobility must be just or else risks not taking place. The Commission will therefore ensure that possibilities under the just transition mechanism are fully explored to make this new mobility affordable and accessible in all regions and for all passengers including those with disabilities and reduced mobility.	justice	affordability, people with disabilities, reduced mobility
2.2 We need to make sustainable alternatives widely available to enable better modal choices	11, 12	47	Despite longstanding policy commitments for fair and efficient pricing in transport, progress has been limited. The ‘polluter pays’ and ‘user pays’ principles need to be implemented without delay in all transport modes. Their environment-related external costs alone amount to EUR 388 billion each year. By internalising these external costs, those who use transport will bear the full costs rather than leaving others in our society to meet them and this will trigger a process towards having more sustainable transport modes with lower external costs.	justice	affordability, external costs
2.1 We need to make all modes of transport more sustainable	4	13	[...] through the increased supply of zero-emission vehicles, will make sustainable mobility more affordable for all.	justice	affordability
2.2 We need to make sustainable alternatives widely available to enable better modal choices	8	35	Active transport modes, such as cycling, have seen growth with cities announcing over 2300 km of extra cycling infrastructure. This should be doubled in the next decade towards 5000 km in safe bike lanes.	policy measures/actions	active travel
1 OUR VISION	1	1	Mobility and transport matters to us all. From daily commuting to work, visiting family and friends, tourism, to the proper functioning of global supply chains for the goods in our shops and for our industrial production, mobility is an enabler of our economic and social life.	purpose/framing	

2.2 We need to make sustainable alternatives widely available to enable better modal choices	11	Flagship 5	FLAGSHIP 5 – PRICING CARBON AND PROVIDING BETTER INCENTIVES FOR USERS	policy measures/actions	
3 SMART MOBILITY – ACHIEVING SEAMLESS, SAFE AND EFFICIENT CONNECTIVITY	20	Flagship 9	FLAGSHIP 9 – MAKING MOBILITY FAIR AND JUST FOR ALL	justice	
6 CONCLUSIONS	24	110	The sustainable European transport system that the EU strives for must be smart, flexible and adaptable to ever-changing transport patterns and needs, based on cutting-edge technological advancements to provide seamless, safe and secure connectivity to all European citizens.	vision, justice	road safety

Table S2 Mentions of issues related to justice and equity in Finland's National Transport System Plan

**Chapter**	**Section**	**Page**	**Citation [2]**	**Category**	**Keywords**
1 General premises	1.1 Role of the transport system in society	8	The transport system plays a very important role in terms of the functioning of society. According to Government Report No. 8/2018 (VNS 8/2018 vp), the societal goals of transport system development include promoting Finland's competitiveness, combating climate change, and regional vitality and accessibility. Transport system planning is a means of advancing these societal goals while also responding to the various mobility and transport needs of customers, i.e. people and businesses, in different parts of Finland.	purpose/framing	climate change, accessibility, needs, businesses
1 General premises	1.1 Role of the transport system in society	8	Development of the transport system is essential in terms of regional development and land use, business competitiveness, combating climate change, good everyday life, security of supply, etc.	purpose/framing	regional aspects, land use, climate chage, everyday life, businesses
1 General premises	1.1 Role of the transport system in society	8	Transport system development efforts also take account of the requirements of preparedness, readiness and emergency conditions.	purpose/framing	resilience, preparedness

1 General premises	1.2 Legal basis for drafting the National Transport System Plan	8	According to section 22 of the Constitution of Finland (731/1999), the public authorities must guarantee the observance of basic rights and liberties and human rights. Adopting a more durable and predictable approach to transport system planning can improve the promotion of realisation of several fundamental rights and freedoms, especially freedom of movement and freedom to engage in commercial activity.	purpose/framing	freedom of movement, commercial activity
1 General premises	1.2 Legal basis for drafting the National Transport System Plan	10	The Plan's drafting process is based on the mobility and transport needs of people and businesses and development of relevant information. [...] The Plan promotes the realisation of freedom of movement as laid down in section 9 of the Constitution such that its measures will particularly improve access to regions and services both in Finland and internationally. The Plan's measures will also improve people's ability to choose different modes of mobility making use of various transport services.	purpose/framing	needs, businesses, freedom of movement, multimodality
1 General premises	1.2 Legal basis for drafting the National Transport System Plan	10	The Plan also promotes the realisation of freedom to engage in commercial activity as laid down in section 18 of the Constitution such that it will require the transport administration to prioritise effective maintenance and development of transport connections in its operations, particularly to meet the needs of businesses and commuters. This will improve people's opportunities to make a living from their chosen work, occupation or business.	purpose/framing	commercial acitivity, needs, businesses
1 General premises	1.2 Legal basis for drafting the National Transport System Plan	10, 11	Under section 20 of the Constitution, the public authorities must endeavour to guarantee for everyone the right to a healthy environment and the possibility to influence the decisions that concern their own living environment. The Plan promotes these rights and freedoms by means such as enhancing the attractiveness of the most environmentally sustainable modes of mobility, thus reducing harmful environmental emissions from mobility.	externalities	health, emissions
1 General premises	1.2 Legal basis for drafting the National Transport System Plan	11	According to the Plan, efforts will be made to develop information on the transport system and the effects of measures taken on the system so as to improve people's opportunities to exercise their participation rights.	justice	participation rights

1 General premises	1.3 Relation of national transport system development to other legislation	12	The United Nations Convention on the Rights of Persons with Disabilities (Finnish Treaty Series 27/2016) includes provisions on accessibility, requiring States Parties to take appropriate measures to ensure people with disabilities access, on an equal basis with others, to the physical environment, to transportation, to information and communications. The purpose of the Convention is to promote, protect and ensure the full and equal enjoyment of all human rights and fundamental freedoms by all people with disabilities. Its key principles include non-discrimination and accessibility. One of the fundamental rights under the Convention involves participation and inclusion of people with disabilities in all decision-making processes concerning them.	justice	people with disabilities, accessibility, participation rights, reduced mobility
1 General premises	1.3 Relation of national transport system development to other legislation	12, 13	By virtue of their self-government, the Saami as a people have the right to decide on their political conditions and promote their own economic, social and cultural development.	justice	Sámi people
1 General premises	1.4 International and EU law dimensions	13	In terms of national transport system planning, the most relevant international agreement concerning the environment is the UN Paris Agreement on climate change (Finnish Treaty Series 75/2016).	vision	climate change
1 General premises	1.5 Links to other decisions and strategies and delimitations of scope	15	The National Transport System Plan was drafted taking account of Finland's regional development priorities for 2020–2023.	vision	regional aspects
1 General premises	1.5 Links to other decisions and strategies and delimitations of scope	15	The measures included in the National Transport System Plan will also promote traffic safety. Traffic safety is covered more extensively in all modes of transport in the national transport safety strategy, which is currently being prepared under the leadership of the Ministry of Transport and Communications.	vision	traffic safety

2 Current state of the transport system and changes in the operating environment	2.1 Current state of the transport system	17	Almost 90% of Finns live within reach of urban centres, which keep expanding further.	current state	urbanisation, cities
2 Current state of the transport system and changes in the operating environment	2.1 Current state of the transport system	17	A passenger car is the most common mode of mobility in people's own residential districts, except for the Helsinki Metropolitan Area, where trips are made on foot slightly more often than by car.	current state	mode share
2 Current state of the transport system and changes in the operating environment	2.1 Current state of the transport system	18	The transport network's condition and daily service levels have significant effects on society. Finland's busy transport routes are mostly in good condition. Traffic fluidity on main roads is good, with the exception of access and ring roads in the largest urban sub-regions, which have seen increasing levels of congestion. On the low-grade road network, daily travel and transport flows are fairly fluid. The condition of low-volume roads and railways has deteriorated.	current state	transport network, congestion
2 Current state of the transport system and changes in the operating environment	2.1 Current state of the transport system	18	With the exception of road traffic, traffic safety is at a good level in Finland. The downward trend in road traffic fatalities stagnated in the late 2010s.	current state	traffic safety
2 Current state of the transport system and changes in the operating environment	2.1 Current state of the transport system	18	Finland's domestic transport emissions have not decreased significantly from 1990 levels. In addition to choices of propulsion systems, cutting down transport emissions will particularly require reductions in passenger car traffic. However, passenger road transport is expected to continue growing at quite a steady rate.	current state, projections, avoid	emissions

2 Current state of the transport system and changes in the operating environment	2.2 Changes in the operating environment	19	Finland’s internal migration flows have gravitated towards urban sub-regions for a long time now. In addition to internal migration, the focus of natural population growth has shifted to large urban sub-regions, which is further intensifying geographical demographic imbalance.	current state, projections	migration
2 Current state of the transport system and changes in the operating environment	2.2 Changes in the operating environment	19	The ageing population will influence traffic behaviour and mobility habits, which will need to be taken into account in transport system planning.	justice	aging population
2 Current state of the transport system and changes in the operating environment	2.2 Changes in the operating environment	19	Inward urban growth is reflected in the growing share of populations and jobs in urban centres and their peripheral and public transport zones. It is fair to assume that commutes will continue to lengthen, particularly in rural areas.	current state, projections	rural areas
2 Current state of the transport system and changes in the operating environment	2.2 Changes in the operating environment	19	Moving forward, multi-local living and increasing remote work may also create new kinds of transport and communications needs.	vision, projections	multi-locality, remote work
2 Current state of the transport system and changes in the operating environment	2.2 Changes in the operating environment	19	Climate change mitigation is one of the most critical challenges. Reduction of greenhouse gas emissions from transport plays a significant role in this respect. Transport system development needs to take account of aspects such as increasing use of alternative propulsion systems and advancements in vehicles and technologies in all modes of transport. Adapting to climate change will require transport system stakeholders to increase awareness of weather and climate risks, for example.	vision	climate change, electrification, risks

2 Current state of the transport system and changes in the operating environment	2.2 Changes in the operating environment	19	Transport and communications are closely connected to the change introduced by increasing digitalisation in society. Technological advancements will heavily influence the transport sector's development for decades to come.	vision	digitalisation
2 Current state of the transport system and changes in the operating environment	2.2 Changes in the operating environment	19	Mobility services are developing towards an increasing variety of options, offering all user groups flexible, efficient, accessible and low-emission mobility services. Demand for accessible services will increase, especially as the population ages. Developments in automation will also open up new opportunities for organising mobility services.	vision, justice	mobility services, aging population, automation, accessibility, population groups
2 Current state of the transport system and changes in the operating environment	2.2 Changes in the operating environment	20	The coronavirus crisis may also bring about longer-term changes in people's mobility habits and patterns. Growth in remote work and multi-local living may contribute to lower passenger transport performance, but some of the changes may also be harmful from the perspective of sustainable development.	vision, projections	multi-locality, remote work
2 Current state of the transport system and changes in the operating environment	2.2 Changes in the operating environment	20	As provision of public transport is highly dependent on public sector support, there is a risk that deteriorating general government finances will also result in declining funding and service levels in public passenger transport.	vision, projections	public transport, funding
2 Current state of the transport system and changes in the operating environment	2.3 Regional characteristics	21	The Saami people's rights and needs must be taken into account in transport system development in Northern Finland.	justice	Sámi people
2 Current state of the transport system and changes in the operating environment	2.4 Finland as part of the international transport system	25	Rail traffic is unevenly distributed in geographical terms. The majority of passenger transport is concentrated in the Helsinki sub-region and Southern Finland. Of the total of about 76 million rail journeys, 94% are made within a 300-kilometre radius from Helsinki, including all commutes and 64% of long-distance journeys.	current state	regional aspects

2 Current state of the transport system and changes in the operating environment	2.5 Transport system funding and existing transport network financing models	31	Transport infrastructure use is taxed moderately in Finland. Unlike almost all the other EU Member States, Finland does not levy any charges on road traffic.	current state	taxation
3 Vision for transport system planning for 2050 – towards a sustainable and accessible Finland	-	33	In 2050, Finland's transport system will function in a way that is environmentally, socially and economically sustainable, ensuring sufficient accessibility for people and businesses. Transport will function in a multimodal and emission-free manner. Mobility and logistics costs will have decreased.	vision, justice	accessibility, businesses, affordability, social justice
3 Vision for transport system planning for 2050 – towards a sustainable and accessible Finland	-	33	The transport system will have been designed taking account of security of supply and the requirements of preparedness and readiness such that Finland will be able to rely on its year-round performance and resilience in all conditions.	vision	resilience, preparedness
3 Vision for transport system planning for 2050 – towards a sustainable and accessible Finland	-	33	The digital transformation will have swept through the transport system, improving its safety and efficiency while offering better services for people and businesses.	vision	digitalisation, businesses, road safety
3 Vision for transport system planning for 2050 – towards a sustainable and accessible Finland	-	33	The transport system will be accessible and equal to all user groups. People and businesses will be mainly satisfied with the transport system. The transport system will have created the prerequisites for punctual and predictable transport services that meet the needs of both people and businesses.	justice	accessibility, equality, businesses, population groups
3 Vision for transport system planning for 2050 – towards a sustainable and accessible Finland	-	33	Traffic safety will be at a high level in all modes of transport and no-one will need to die or sustain serious injuries in traffic.	vision	traffic safety

3 Vision for transport system planning for 2050 – towards a sustainable and accessible Finland	-	33, 34	Commuters will mainly use sustainable modes of mobility within cities and commuting areas and between urban sub-regions. As a result of infrastructure and service development, public transport will form the backbone of sustainable transport. Rail transport development will have efficiently enabled sustainable community structures in the largest urban sub-regions and along the routes between them. A significant proportion of knowledge workers will have shifted to working remotely while remote technologies will also be widely used in various services. Trips made on foot or by bicycle will have replaced those made by passenger car to a considerable extent, particularly in urban sub-regions. Use of new versatile mobility and transport services will have become an established feature of everyday life.	vision	cities, commuters, public transport, remote working, activie travel, mobility services
3 Vision for transport system planning for 2050 – towards a sustainable and accessible Finland	-	34	People living in other urban and sparsely populated areas will have access to mobility services offering a better service level to meet their needs.	vision	mobility services, cities, rural areas
3 Vision for transport system planning for 2050 – towards a sustainable and accessible Finland	-	34	Passenger car use will have become more sustainable with the development of lower-emission propulsion systems and vehicle technologies. [...] People will be able to use fossil-free propulsion systems throughout the country.	vision	electrification, regional aspects
4 Objectives of the National Transport System Plan and their specific strategic guidelines	4.1 Objectives of the Plan	35	The following three parallel objectives were set for the National Transport System Plan, all striving to mitigate climate change:<br>Accessibility: The transport system will ensure access to the whole of Finland and will respond to the needs of business, employment and housing.<br>Sustainability: Opportunities to choose more sustainable modes of mobility will improve – particularly in urban sub-regions.<br>Efficiency: The socio-economic efficiency of the transport system will improve.	vision	climate change, accessibility

4 Objectives of the National Transport System Plan and their specific strategic guidelines	4.2 Specific strategic guidelines for the Plan's objectives	36	2) Interregional accessibility<br>Significant business and commuting connections will be developed between regional centres and between Helsinki and the rest of Finland. Average travel times between cities with significant commuting or other travel activities will become shorter. Public transport's competitiveness relative to passenger car use will improve on key commuting links between regional centres.	policy measures/actions	regional aspects, commuting, businesses
4 Objectives of the National Transport System Plan and their specific strategic guidelines	4.2 Specific strategic guidelines for the Plan's objectives	37	3) Intraregional accessibility<br>Important business and commuting connections will be preserved between regional peripheries and regional and other key centres. [...] The current coverage of the road network will be secured in order to meet the needs of inhabitants.	policy measures/actions	regional aspects, commuting, businesses
4 Objectives of the National Transport System Plan and their specific strategic guidelines	4.2 Specific strategic guidelines for the Plan's objectives	37	4) Travel and transport service levels<br>Satisfaction with the transport system will improve among all population groups and businesses.<br>The effectiveness of transport nodes and travel chains will be developed (transport services, information, other services).<br>Mobility opportunities will be ensured for different population groups to guarantee social sustainability.<br>The transport network will support and promote sustainable community structures. The transport network will only be expanded if this promotes sustainable structures.	justice	needs, land use , population groups, social justice
4 Objectives of the National Transport System Plan and their specific strategic guidelines	4.2 Specific strategic guidelines for the Plan's objectives	37	Road safety will be improved.	policy measures/actions	road safety
4 Objectives of the National Transport System Plan and their specific strategic guidelines	4.2 Specific strategic guidelines for the Plan's objectives	37, 38	Strategic guidelines for sustainability<br>Sustainable modes of mobility will be promoted by a wide variety of means, particularly in urban sub-regions with sufficient populations to allow emission reductions to be achieved cost-efficiently. Sustainable modes of mobility and transport will also be developed outside urban sub-regions.<br>[...]<br>The shares of public transport, walking, cycling and other sustainable modes of mobility will increase while greenhouse gas emissions from transport will decrease, contributing to the achievement of the climate target.	policy measures/actions	sustainable modes, cities, rural areas, mode shift, public transport, active travel

4 Objectives of the National Transport System Plan and their specific strategic guidelines	4.2 Specific strategic guidelines for the Plan's objectives	38	Strategic guidelines for efficiency<br>The existing transport network will be put to maximum use while implementing the most efficient and effective measures to close any gaps.<br>[...]<br>New transport investments will promote sustainable transport and their social benefits will exceed the costs involved.<br>[...]<br>Measures concerning services will be targeted so as to achieve the highest possible level of emission reductions and/or accessibility relative to the costs involved.	justice	social justice, accessibility, efficiency, external costs

References

[1] European Commission. 2020. Communication from the Commission to the European Parliament, The Council, The European Economic and Social Committee and the Committee of the Regions, Sustainable and Smart Mobility Strategy – putting European transport on track for the future, COM/2020/789 final. https://eur-lex.europa.eu/legal-content/EN/TXT/?uri=CELEX%3A52020DC0789

[2] Ministry of Transport and Communications. 2021. The National Transport System Plan for 2021–2032. Publications of the Finnish Government 2021:77. http://urn.fi/URN:ISBN:978-952-383-804-8
